# An Electrotype

**Published:** 1855-02

**Authors:** 


" An Electrotype.—The Patterson (N. J.) Intelligencer gives a curious incident of the late thunder storm.

"A little girl was standing at a window before which was a young maple tree. After a brilliant flash of lightning, a complete image of the tree was found imprinted on her body. This is not the first instance of the kind, but it is a singular phenomenon."—*Public Ledger.*

[Dr. ROBERT JOHNS, of Dublin, on SYPHILIS as a cause of Abortion and Premature Labor, after referring to several cases, draws the following deductions:]

1. That secondary syphilis is not curable in the pregnant female.

2. That mercury is the only certain means of preventing the abortions and premature labors depending on syphilis.

3. That both parents must be submitted to this treatment.

4. That as the disease in such cases exists in the secondary form, the system must be kept under the influence of the metal for at least six weeks, and in some cases even longer.

5. That syphilis is communicable to the female through the semen of the male, without the presence of any ulcer or purulent discharge.

6. That secondary ulcers on the female genitals do not infect the male by whom she was contaminated, so long as he is poisoned by the infection which gave it to her.

7. That as syphilis is communicable from a child to its nurse, and *vice versa.* great care ought to be taken that an infected child be not given to a sound nurse, nor a pocky nurse be hired for a sound child.

8. That syphilis in infants is only curable by mercury, which is best given directly to the child, as also to its nurse.

9. That there is no disease of the uterus, save the Hunterian chancre, which is pathognomonic of syphilis.

10. That the order of the abortions and premature labors is a very good test of their cause.

11. That ulceration may exist to a great amount on the os and cervix uteri, and not be discoverable by the toucher.

12. That the patulous state of the os uteri, induced by inflammation, may be present during pregnancy.

13. That children dying in the uterus from syphilis, like those dying from other causes, are thrown off within a fortnight or so after the cessation of vitality.

14. That there is a class of abortions and premature confinements preventible by iodide of potassium and similar medicines.—*Dublin Quar. Jour. of Med. Science.*

